# Perceptions of Hunting and Hunters by U.S. Respondents

**DOI:** 10.3390/ani7110083

**Published:** 2017-11-04

**Authors:** Elizabeth Byrd, John G. Lee, Nicole J. Olynk Widmar

**Affiliations:** 1 Natural Resources, and Design, Davis College of Agriculture, West Virginia University, Morgantown, WV 26506, USA; ebyrd@mail.wvu.edu; 2 Department of Agricultural Economics, Purdue University, West Lafayette, IN 47907, USA; jlee1@purdue.edu

**Keywords:** animal welfare, hunting, hunting practices, public acceptance, public perception

## Abstract

**Simple Summary:**

An online survey of 825 U.S. residents was conducted to determine their views on hunting, hunters, and hunting practices within the United States. Overall, 87% of respondents agreed that it was acceptable to hunt for food. However, only 37% agreed that it was acceptable to hunt for a trophy. Those who knew hunters, participated in hunting-related activities, or visited fairs or livestock operations had more favorable opinions on hunting or hunters.

**Abstract:**

Public acceptance of hunting and hunting practices is an important human dimension of wildlife management in the United States. Researchers surveyed 825 U.S. residents in an online questionnaire about their views of hunting, hunters, and hunting practices. Eighty-seven percent of respondents from the national survey agreed that it was acceptable to hunt for food whereas 37% agreed that it was acceptable to hunt for a trophy. Over one-quarter of respondents did not know enough about hunting over bait, trapping, and captive hunts to form an opinion about whether the practice reduced animal welfare. Chi-square tests were used to explore relationships between perceptions of hunters and hunting practices and demographics. Those who knew hunters, participated in hunting-related activities, visited fairs or livestock operations, or were males who had more favorable opinions on hunting. A logistic regression model showed that not knowing a hunter was a statistically significant negative predictor of finding it acceptable to hunt; owning a pet was statistically significant and negative for approving of hunting for a trophy.

## 1. Introduction

Maintaining public support for hunting is important to wildlife management practices in the United States [1]. In the United States, the revenue from hunting licenses provides revenue for conservation and there is no commercial trade in wildlife. U.S. wildlife managers appreciate the importance of understanding the broader public’s interest in wildlife policy issues and public attitudes towards wildlife [2,3,4,5,6]. Similarly, previous research has focused on hunters’ perceptions of wildlife disease and wildlife management activities [7]. Hunting is an important wildlife management tool that is often thrust into the national media. In 2013, Time Magazine featured wildlife population management on the cover and generally portrayed hunting as a valuable way to manage some wildlife populations [8]. On the other hand, a massive public outcry resulted in a teenager’s hunting photos being removed from Facebook even though those animals were harvested outside the United States [9]. Similarly, there was a great deal of media coverage about a failed Maine ballot initiative aimed at ending the use of dogs, traps, and bait in black bear hunts [10]. Such stories have the potential to sway public opinion against hunting, hunters, and hunting practices. In addition, the public expects to provide input on wildlife issues in the United States [11]. When the U.S. public feels that their interests have not been met, they may resort to other means such as court cases, administrative appeals, and ballot initiatives [3]. Further, the public and wildlife agency employees have been found to have differing levels of support for the lethal management of wildlife [12]. Therefore, it is important to study public perception of wildlife management, in this case hunting, because wildlife managers cannot rely on their own support for the lethal management of wildlife. Public support is necessary for hunting to continue [13]. Without hunting, wildlife management in the United States would suffer a loss of both economic and social support [14]. 

Hunting is an important component of wildlife management because it provides funds for and is an efficient means of controlling wildlife populations [15]. Furthermore, hunting is the primary management tool for species such as the white-tailed deer [16]. According to the National Survey of Fishing, Hunting, and Wildlife-Associated Recreation, during 2011, 6% of U.S. residents 16 and older participated in hunting with a total of 13.7 million hunters [17]. Stedman and Decker [18] pointed out that non-hunters are also hunting stakeholders. Their research showed that many non-hunters in New York State held positive beliefs about hunting and one-third held pro-wildlife management beliefs despite not being hunters themselves [18]. 

Hunting is culturally important for millions of Americans [19]. Hunting is typically viewed as a “rural” activity [19]. Urbanization has been identified as a prominent demographic factor dampening overall participation in hunting [19]. Social relationships with hunters or participation in hunting-related activities may socialize non-hunters to have similar beliefs as hunters [18]. Support for hunting can vary based on the purpose of the hunt [2,20].

Additional information about the social acceptability of wildlife management techniques, including hunting, is needed to better enable decision making by wildlife officials. The primary goal of this research was to quantify U.S. resident perceptions of hunters, hunting, and hunting practices on a nationwide scale. The secondary goal of the research was to explore the demographics and other characteristics related to those perceptions. It is hypothesized that sentiments towards hunters and hunting are associated with age and gender. Furthermore, it is hypothesized that respondents who are familiar with hunters (i.e., know someone who hunts) will be more accepting of hunting, as will those who have more experience with livestock operations or fairs. 

## 2. Methods

### 2.1. Nationwide Survey of U.S. Residents

An online survey was administered in November of 2014 to respondents recruited from a large opt-in panel maintained by Lightspeed, GMI, a global market research organization. An opt-in panel is one where potential respondents opt-in or voluntarily sign up to be panel members. The panel provider was able to target panel members by their demographic information to reach desired targets, such as being representative of the U.S. population based on the U.S. Census [21]. The sample was targeted to be representative of the U.S. population for gender, age, household income, education level, and region of residence, according to the U.S. Census [21]. Respondents were required to be at least 18 years of age to participate in the survey. Respondents could only participate if contacted by Lightspeed-GMI and provided the survey link. Respondents were not permitted to take the survey more than once. The survey was hosted on the Qualtrics platform at Purdue University. A total of 825 U.S. residents completed the survey.

Respondents were asked about demographics, relationships with animals in general, and questions about familiarity with hunting and hunters, perceptions of hunting and hunting practices, pet ownership, participation in fairs (e.g., state or county), and visiting a livestock operation were asked. For the purposes of this analysis, reporting having visited a dairy, pig, or beef farm were aggregated to represent having visited a livestock operation. Previous research included pet ownership in a study about the perceptions of the welfare of livestock animals [22,23,24]. Limited research exists that links pet ownership to perceptions of wild animal welfare. If pet ownership and knowledge of livestock are linked to sentiments towards domestic animal welfare, then it is likely that these are also linked to hunting and wild animal welfare.

In addition to questions about familiarity with animals in general, respondents were asked a series of questions to elicit their relationship to hunters and participation in hunting and hunting-related activities. Social relationships with hunters, such as friends or relatives who hunt, or participating with these individuals in hunting-related activities may socialize non-hunters to have similar beliefs as hunters [18]. Specifically, Stedman and Decker [18] included non-hunters’ relationships to hunters and participation in hunting-related activities. In the present study, it was hypothesized that respondents who had hunted themselves, had friends or family who were hunters, or had participated in hunting-related activities would regard hunting more favorably. In order to gauge familiarity with hunters and hunting, respondents were asked “Do you know anyone who hunts?” and were permitted to select all responses that applied to them. The list included: “I hunt” and “I do not know anyone who hunts”. 

In addition to asking respondents if they knew hunters, the survey also contained questions that asked if respondents had participated in activities related to hunting that did not require pursuing game [18]. It was therefore hypothesized that respondents that had participated in hunting-related activities would have more favorable opinions of hunters and hunting. Respondents were asked to respond “Yes” or “No” to the question “Have you participated in any of the following activities?” for a number of activities which were adapted from Stedman and Decker [18] including, eat game meat obtained through hunting, participate in target shooting, and helped a hunter look for signs of wildlife in preparation for hunting. 

Respondents were asked if they agreed with a variety of reasons that people hunt. The reasons were to obtain food, trophy hunting, wildlife population control, reduce predator population, and control crop damage. Respondents could select agree or disagree. Respondents were also asked a series of questions about their beliefs and sentiments towards hunting and hunters. Respondents could select agree, neither agree nor disagree, or disagree in response to a series of statements regarding sentiments towards hunting such as “Hunting is an important rural tradition.” Most of these statements tested were included in either Ljung et al. [1] or Stedman and Decker [18]; some statements were included in both studies. The final two statements were developed by researchers in the current study. The survey was approved by the local university’s institutional review board (IRB Protocol Number 1410015306).

### 2.2. Data Analysis

Pearson’s Chi-Square test was used to examine relationships between demographics, hunting participation, and attitudes towards hunting and hunters. In addition, two logistic regressions were used to determine which demographics were statistically significant predictors of whether the respondent agreed with hunting for food and trophy hunting. A logit model was constructed based on the question “Please indicate whether you agree or disagree with the following reasons people hunt”. For the purposes of this analysis, the categories of “disagree” and “neither agree nor disagree” were aggregated. Thus, the logit model showed the factors that were associated with agreeing with a reason to hunt, such as hunting for food. 

## 3. Results

### 3.1. Summary Statistics

The mean age of respondents was 47 years and the sample was composed of 51% (*n* = 421) female and 49% (*n* = 404) male respondents. Overall, 48% (*n* = 401) of households had an annual pre-tax income of less than $40,000, 35% (*n* = 289) fell into the $40,000–$79,000 range, 11% (*n* = 87) reported income of $80,000–$119,000, and 6% (*n* = 48) reported an annual pretax income of $120,000 or more. Overall, 97% (*n* = 800) of respondents graduated high school and 33% (*n* = 272) had earned at least a bachelor’s degree. Nationwide, 86% of residents have graduated high school and 29% have attained at least a bachelor’s degree (U.S. Census, 2014). Overall, 65% (*n* = 536) of households reported owning a pet (defined as owning at least one cat or dog). Sixty-seven percent (*n* = 553) reported having ever visited a fair and 53% (*n* = 437) reported having visited a livestock operation. 

### 3.2. Connections to Hunting

Forty-seven percent (*n* = 388) of respondents reported that they had eaten game meat obtained through hunting at some point in their lifetime. Thirty-seven percent of respondents had participated in target shooting and 20% (*n* = 165) of survey-takers stated they had helped a hunter look for signs of wildlife in preparation for hunting. While 36% (*n* = 297) of respondents did not know anyone who hunts, 64% (*n* = 528) of respondents knew a hunter. Fourteen percent of respondents stated they hunted. Of those who reported they hunted, 73% were male and 27% were females. Ten percent (*n* = 83) of respondents were males who hunted and were females who hunted. 

### 3.3. Sentiments Toward Hunting and Hunters

Respondents were asked if they agreed or disagreed with a series of statements about the role of hunting and their sentiments regarding hunting and hunters. Twenty-nine percent (*n* = 239) of respondents agreed that hunters should be able to post pictures of the game they harvest on social media; however, 26% (*n* = 215) disagreed. Overall, 69% (*n* = 569) of respondents agreed that everyone who hunts should take a hunter safety course. More than one third agreed that hunting reduced the chance of animal vehicle collisions, but 21% disagreed (*n* = 173). Forty-five percent (*n* = 371) of respondents agreed that hunting helped to reduce damage to agricultural crops and 31% (*n* = 256) agreed that it helped reduce wildlife diseases by reducing animal populations. Overall, 31% (*n* = 256) agreed that hunting provides funds used to manage other species of wildlife that are not hunted. Thirty-five percent (*n* = 289) agreed that the demand for hunting maintains wildlife habitats, while 51% (*n* = 421) agreed hunting is an important rural tradition. Forty-eight percent (*n* = 396) of respondents agreed that hunting helps to keep nature in balance. On the other hand, 39% (*n* = 322) of respondents agreed that hunting commonly results in species becoming threatened or endangered and 37% (*n* = 305) of respondents agreed that hunters often ignore safety rules. Finally, one third of respondents regarded sport or recreational hunting as cruel to animals. 

In the interest of brevity, values representing the percentage of individuals in each category who did not agree with the statement have been omitted in the Chi squared results tables (Table 1 and Table 2). Because the values in each column must necessarily sum to 100, omitted variables can be calculated. For example, 51% of those age 45 and over agreed with the statement “Hunting helps keep nature in balance”, and 49% of respondents age 45 and over did not agree (selected neutral or disagree) with the same statement. 

Respondents who agreed that hunting helps keep nature in balance more frequently reported being older (45 or more years old), having visited a livestock operation, and having visited a fair (Table 2). Results were similar for agreeing that hunting was an important rural tradition, but respondents agreeing with this statement also more frequently reported being male. Older (45 years of age or more) female respondents more frequently agreed that hunters often ignore safety rules. Respondents who were 45 or more years old were more likely to agree that hunting helps control wildlife diseases, reduces agricultural damage, reduces vehicle collisions, and every hunter should have taken a hunters’ safety course. Male respondents were more likely to agree that hunting provides funds to manage other species, reduce agricultural damage, reduce vehicle collisions, and hunters should be able to post pictures on social media. Finally, respondents who agreed that hunting maintains wildlife habitats, hunting helps control wildlife diseases, hunting provides funds to manage other species, hunting helps to reduce agricultural damage, hunting helps to reduce vehicle collisions, every hunter should have taken a hunters’ safety course, and hunters should be able to post pictures on social media more frequently reported having visited a livestock operation or fair.

Of those who agreed with the statements that hunting helps keep nature in balance, is an important rural tradition, maintains demand for habitat, helps reduce wildlife disease, provides funds for other wildlife species, reduces agricultural damage, and reduces vehicle collisions, those respondents more frequently reported being hunters or knowing a hunter (Table 2). Similar results were found for participating in hunting-related activities. Of those who agreed that hunters should be able to post pictures of wild animals they have hunted, respondents more frequently reported being a hunter, knowing a hunter, and participating in hunting-related activities. 

With regard to the remaining statements, those who agreed that any kind of sport or recreational hunting is cruel to animals more frequently reported not having eaten game meat obtained through hunting. Among those who agreed that hunters often ignore safety rules, respondents were more frequently non-hunters and had not eaten game meat or participated in target shooting. Respondents who agreed that hunting commonly results in a species becoming threatened more frequently identified themselves as not hunting, not knowing any hunters, not having eaten game meat, and not having participated in target shooting.

### 3.4. Acceptable Reasons to Hunt

When asked to characterize reasons to hunt as either acceptable or unacceptable, 87% of respondents agreed with hunting to obtain food which is the most widely accepted reason included in the survey. The next most acceptable reasons to hunt were for wildlife population control with 72% of respondents selecting agree, followed by 66% that agreed with hunting to reduce a predator population. Sixty-three percent of respondents agreed with hunting to control crop damage. However, only 37% of respondents agreed with trophy hunting being acceptable.

Of those respondents who agreed with each reason, respondents were more frequently male than female (Table 3). Similarly, those respondents who agree with each use more frequently reported having visited a livestock operation than not (Table 4). With the exception of trophy hunting, those respondents who agreed with the acceptability with each of the reasons surveyed more frequently reported having visited a fair (Table 3). Among those who agreed with the six reasons to hunt, respondents reported knowing a hunter (or hunted themselves) more frequently than not and more frequently reported having participated in all of the hunting-related activities included (Table 4).

The logistic regression model (Table 5) showed that not knowing a hunter was a statistically significant negative predictor of finding it acceptable to hunt for both reasons; being a hunter was positively related to agreeing with both reasons. Visiting a fair was a statistically significant and positive predictor of approving of hunting for food. Visiting a livestock operation was a statistically significant and positive predictor of agreement with hunting for a trophy. Owning a pet was statistically significant and negative for approving of hunting for a trophy. Being male contributed to being more likely to approve of hunting for a trophy.

### 3.5. Practices Reducing Welfare of Hunted Animals

At least one-fourth of respondents were unfamiliar with each of the hunting practices studied. Thirty-five percent of respondents indicated that they were unfamiliar with hunting over bait and 32% were unfamiliar with captive hunting. Captive hunting is when the animals are confined in large fenced in areas for hunting rather than running wild. Likewise, 26% were unfamiliar with the practice of hunting with dogs and 25% were unfamiliar with either hunting in wildlife preserves or trapping. Sixty-five percent had formed an opinion (e.g., agree/neutral/disagree) on hunting over bait, 68% formed an opinion on captive hunting, 75% had formed an opinion on hunting in wildlife preserves, 75% formed an opinion on trapping, and 74% had formed an opinion on the use of dogs. 

Respondents who formed an opinion about each of the practices, with the exception of hunting in wildlife preserves, were more frequently under the age of 45 (Table 4). On the other hand, respondents who reported not knowing enough to respond were more frequently 45 years of age or older. This was with the exception of hunting in wildlife preserves, where no statistical difference was found. Respondents who formed an opinion were more frequently male, while those who didn’t know enough to respond were more frequently female for all practices studied (Table 4). Respondents who formed an opinion were more frequently college graduates, while those who didn’t know enough to respond were more frequently not college graduates for all practices (Table 4). Similarly, pet owners, those who had visited a livestock operation, and those who had visited a fair more frequently reported having an opinion. Non-pet owners and those who had not visited a livestock operation or fair more frequently reported not knowing enough to respond than those who owned a pet or visited a livestock operation or fair (Table 5).

## 4. Discussion

Respondents in this survey were slightly more educated than the national average, where 85.7% of Americans over 25 years of age have graduated high school and 28.5% have at least a four-year degree [21]. On the other hand, the respondents had a slightly lower household income than the U.S. median household income of $53,046 [21]. However, the current study features contemporary data from a nationwide survey that includes both hunters and non-hunters. This sample represents the general U.S. public’s perceptions of hunters and hunting, which will prove very valuable to wildlife managers and others who seek to understand the public perception of hunting in the United States. 

Older respondents (45 and older) tended to be more supportive of hunting and hunters; a higher percentage of respondents agreed with statements such as hunting keeps nature in balance, reduces agricultural damage, and reduces vehicle collisions. This is consistent with previous research that found that older individuals were more likely to support hunting [25]. However, a higher percentage of those 45 and older also agreed that hunters often ignore safety rules and every person who hunts should have taken a hunters’ safety course. This indicates that while these respondents recognize the benefits of hunting, they are also concerned about the safety aspects of hunting. 

College graduates more frequently reported having an opinion (e.g., agree, neutral, disagree) when asked about whether a number of hunting practices reduce the welfare of hunted animals. Those who had not graduated college more frequently reported not knowing enough to respond. Previous research has considered differences in the support of hunting based on education level. Teel, Krannich, and Schmidt [26] found that respondents with lower education levels were less opposed to predator management practices for cougar and black bear. Similarly, respondents with less education more frequently approved of a specific moose hunt [3]. These results are further supported by MacKay and Campbell [27], who found that strong supporters of hunting tourism tended to have lower levels of education than those respondents who were classified as moderate or low supporters.

Approximately one quarter of respondents reported not knowing enough about hunting over bait, hunting in wildlife preserves, captive hunts, trapping, and using dogs in hunting to respond to whether the practice reduced animal welfare. This is particularly interesting in light of recent efforts to change the law in the state of Maine to disallow black bear hunting using dogs, trapping, and bait. These efforts were backed by animal welfare organizations. Although this example is localized to Maine, it illustrates the fact that a segment of the public may not have formed an opinion on potentially controversial practices that they are being asked to vote on. Those individuals who have not formed an opinion may seek out information about the practice from various sources. In this case, part of the strategy of wildlife managers may be to educate the public on these specific practices.

One part of the potential explanation for women being less accepting of hunting is that women are less familiar with hunting than men. Women represent a small percentage of hunters and substantial differences exist amongst different groups (or typologies) of female hunters [28]. Furthermore, fewer female hunters could be due to historic gender roles [13]. Overall, hunters are more frequently male [17]; nationwide, 11% of hunters are female [17]. The second part to this explanation is that women tend to view animals and wildlife differently than men. In past research, women were more likely to report concern for animal welfare in general [22], were less supportive than men of lethal means of wildlife management [12], less likely to prefer euthanasia of feral cats than men [29], and more strongly oppose hunting for either recreational or food-gathering reasons [30].

This study included pet ownership and having visited a livestock operation or fair as demographic variables to proxy animal familiarity. Previous research has shown that pet owners were more likely to express concern for the welfare of farmed pigs [22]. In addition to owning pets, the public can also learn about animals by visiting a farm or fair where animals are being raised or exhibited. In fact, the premise underlying agritourism is that by showing the public the process of food animal production, it will lessen visitors’ concerns about animal welfare issues [31]. County and state fairs are common across the rural U.S. and often feature animal related activities such as livestock exhibits, horse shows, pony rides, petting zoos, circuses, and wagon rides. Specific to wildlife management, large fairs often feature exhibits from state departments of fish and wildlife and natural resource agencies. For example, fair goers can interact with wildlife managers, try wild game meat, and purchase their hunting and fishing licenses. It is not surprising that those individuals who had visited a livestock operation or fair had more favorable opinions on hunting. Visitors to fairs and livestock operations may be more likely to be rural residents. Previous research found that hunters are more likely to have grown up in rural areas [32,33], so it may be that residents of rural areas (or those who grew up in rural areas) may be more predisposed be in favor of elements of the rural lifestyle including hunting, fairs, and farms. Therefore, visiting farms/livestock operations or fairs could be useful variables to include in future research. In particular, fairs could be good outlets for public outreach were wildlife managers could reach populations of people who are predisposed to accept hunting. 

One factor clearly associated with positive sentiments towards hunting and hunters is involvement in hunting and knowing hunters. Having other family members who hunt and being exposed to hunting are two factors that initiate individuals into hunting [20]. Respondents who participated in hunting-related activities generally had positive sentiments about hunting and hunters. This is consistent with the finding that hunters and respondents approving of hunting were more likely to support lethal methods of control for coyotes found by Martinez-Espineira [25]. In general, those respondents who reported not knowing anyone who hunts and not having participated in hunting-related activities were less supportive of hunting.

The fact that the majority of respondents agreed with hunting for food was consistent with previous research which found that nearly three-quarters of U.S. residents approved of legal hunting and most Americans support hunting for food or for both food and recreation [20]. However, there is less support for hunting for recreation alone or hunting for a trophy [20,26]. Therefore, one strategy the hunting industry and/or wildlife managers may wish to consider is to highlight the fact that hunting has the potential to provide food, even during trophy and/or captive hunts. 

Most Americans feel hunting safety courses should be mandatory; past research found that 89% of current hunters and 93% of non-hunters are in favor of mandatory hunting education courses for new hunters [20]. In fact, most states require hunter safety courses in order to purchase a license [34]. The current research suggests that even those individuals who are socialized to approve of hunting (by participating in hunting-related activities) are still concerned about the safety aspect of hunting. Therefore, one strategy of wildlife managers could be to highlight the safety aspects of hunting to the public.

## 5. Summary and Conclusions

Hunting is an important wildlife management tool [13]. Understanding contemporary public opinion on hunting, hunters, and hunting practices is important for wildlife managers and policy makers. Social acceptance of hunting could potentially change over time, as could sentiments for various practices and hunting for various reasons. The current research provides a contemporary survey of U.S. resident perceptions of hunting and hunters from a nationwide sample. The results from the current study are consistent with previous research, where being male, being a hunter, knowing a hunter, or participating in hunting-related activities is positively related to having more favorable opinions of hunting, hunters, and hunting practices. Additional demographic variables were included in the current study that proved to be valuable; those who had visited a fair or livestock operation also held more positive opinions on hunting and hunters. 

While respondents may hold positive views on the benefits of hunting, they can still have concerns about hunting safety. For example, respondents 45 years of age and older more frequently agreed that hunting helps keep nature in balance and reduces the risk of vehicle collisions, and also more frequently agreed that hunters often ignore safety rules and every hunter should have taken a hunters’ safety course. The public can simultaneously appreciate the benefits of hunting and have concerns about the safety of hunting. A lack of familiarity with hunting and understanding of hunting’s role in wildlife management could be addressed with education efforts [35]. Consequently, there is room for education programs to address the public’s concern (or lack of knowledge) about the nature of some hunting practices if wildlife managers deem it important.

This research has contributed to the literature by surveying U.S. residents nationwide about their perceptions of hunting and hunters. Caution should be used in interpreting these results for countries other than the U.S. because the system of wildlife management is very different in other countries. Future research could explore the relationship between hunting and visiting fairs and livestock operations as it relates to the rural-urban divide. In addition, further research into the relationships between wildlife value orientations and observable demographics could be explored in light of the additional demographic factors included in this research. Understanding the public’s perception of hunting and hunters is important to wildlife managers to ensure the continued social acceptance of hunting, both generally and with respect to specific practices. Furthermore, understanding the public’s concern will help wildlife managers design programs and policies and communicate with the general public.

## Figures and Tables

**Table 1:** Presentation of statistically significant differences in agreement with statements about hunting by demographic categories (α = 0.05 denoted by “*”; *n* = 825).

Statements	Age		Gender		Pet Owner		Visited a Livestock Operation		Visited a Fair	
	45 and Older	Under 45	Female	Male	No	Yes	Have not Visited	Have Visited	Have not Visited	Have Visited
Hunting helps keep nature in balance.	51 *	44 *	46	50	40	49	37 *	58 *	35 *	55 *
Hunting is an important rural tradition.	57 *	44 *	46 *	56 *	51	51	41 *	60 *	35 *	59 *
I regard any kind of sport or recreational hunting as cruel to animals.	34	31	34	32	28 *	36 *	33	32	30	35
Hunters often ignore safety rules.	41 *	34 *	42 *	33 *	30 *	42 *	38	37	37	38
Hunting commonly results in a species becoming threatened or endangered.	38	40	40	38	37	40	37	40	35	41
Demand for hunting maintains wildlife habitat.	38	32	33	37	34	36	26 *	43 *	27 *	39 *
Hunting help control wildlife diseases by reducing animal populations.	42 *	31 *	36	38	34	39	30 *	43 *	24 *	43 *
Hunting provides funds used to manage other wildlife species that are not hunted.	34 *	48 *	25 *	37 *	30	31	21 *	40 *	20 *	36 *
Hunting helps reduce agricultural damage by reducing animal populations.	53 *	36 *	39 *	51 *	43	46	34 *	55 *	29 *	53 *
Hunting reduces the risk of dangerous vehicle collisions with wildlife by reducing animal populations.	41 *	32 *	33 *	41 *	33	39	27 *	46 *	23 *	44 *
Every person who hunts should have taken a hunters’ safety course.	82 *	55 *	69	70	65 *	72 *	64 *	75 *	50 *	79 *
Users of social media should be allowed to post pictures of the wild animals they have hunted/harvested.	28	31	26 *	34 *	28	31	24 *	35 *	19 *	44 *

Note 1: In the interest of brevity, the values representing the percentage of individuals in each category who disagreed with that statement have been omitted. Because the values in each column must sum to 100, omitted variables can be calculated. For example, 51.3% of those age 45 and over agreed with the statement “Hunting helps keep nature in balance.” Thus, 48.7% of respondents age 45 and over did not agree (selected neutral or disagree) with the statement “Hunting helps keep nature in balance.” Note 2: The numbers appear in bold when there is a statistically significant difference at the 5% level. For example, when reading the “Hunting helps keep nature in balance” row, the first column (45 and older) is significantly different than the second column (under 45) at the 5% level.

**Table 2:** Presentation of statistically significant differences in agreement with statements about hunting and participation in hunting-related activities (α = 0.05 denoted by “*”).

Statement	Respondent Hunts		Respondent Knows a Hunter		Eat Game Meat Obtained through Hunting		Participate in Target Shooting		Help a Hunter Look for Wildlife Sign in Preparation for Hunting	
	No	Yes	Yes	No	Yes	No	Yes	No	Yes	No
Hunting helps keep nature in balance.	44 *	73 *	61 *	25 *	69 *	29 *	65 *	38 *	77 *	41 *
Hunting is an important rural tradition.	47 *	74 *	60 *	34 *	72 *	32 *	69 *	41 *	76 *	45 *
I regard any kind of sport or recreational hunting as cruel to animals.	34	27	31	37	28 *	37 *	32	34	27	34
Hunters often ignore safety rules.	39 *	27 *	35	41	33 *	42 *	32 *	41 *	32	39
Hunting commonly results in a species becoming threatened or endangered.	40 *	29 *	35 *	46 *	34 *	43 *	34 *	42 *	34	40
Demand for hunting maintains wildlife habitat.	31 *	58 *	44 *	19 *	51 *	21 *	49 *	27 *	65 *	28 *
Hunting help control wildlife diseases by reducing animal populations.	34 *	56 *	46 *	21 *	53 *	23 *	51 *	29 *	60 *	31 *
Hunting provides funds used to manage other wildlife species that are not hunted.	27 *	53 *	39 *	16 *	47 *	16 *	49 *	20 *	58 *	24 *
Hunting helps reduce agricultural damage by reducing animal populations.	41 *	66 *	54 *	28 *	65 *	27 *	62 *	35 *	70 *	38 *
Hunting reduces the risk of dangerous vehicle collisions with wildlife by reducing animal populations.	34 *	55 *	46 *	21 *	53 *	23 *	51 *	28 *	60 *	31 *
Every person who hunts should have taken a hunters’ safety course.	69	73	74 *	61 *	75 *	65 *	75 *	66 *	70	69
Users of social media should be allowed to post pictures of the wild animals they have hunted/harvested.	27 *	48 *	36 *	17 *	44 *	17 *	44 *	21 *	54 *	23 *

Note 1: In the interest of brevity, the values representing the percentage of individuals in each category who disagreed with that statement have been omitted. Because the values in each column must necessarily sum to 100, omitted variables can be calculated. For example, 43.6% of those who did not hunt agreed with the statement “Hunting helps keep nature in balance.” Thus, 56.4% of respondents who did not hunt did not agree (selected neutral or disagree) with the statement “Hunting helps keep nature in balance.” Note 2: The numbers appear in bold when there is a statistically significant difference at the 5% level. For example, when reading the “Hunting helps keep nature in balance” row, the first column (No) is significantly different than the second column (Yes) at the 5% level.

**Table 3:** Presentation of statistically significant differences in hunting acceptance across demographic categories (α = 0.05 denoted by “*”).

Statement	Reasons/Choices	Age		Gender		Education		Pet Ownership		Visited a Livestock Operation		Visited a Fair	
		45 And Older	Under 45	Female	Male	Did not Graduate College	College Graduate	Non Pet Owner	Pet Owner	Have not Visited	Have Visited	Have not visited	Have Visited
Finds Reason to Hunt Acceptable													
Finds Reason Acceptable	Obtain Food	88	85	86	88	80	87	86	87	83 *	90 *	79 *	91 *
	Trophy Hunting	63	37	31 *	43 *	38	36	38	36	32 *	42 *	32	39
	Wildlife Population Control	74	69	69 *	75 *	68	73	72	71	66 *	77 *	63 *	76 *
	Reduce Predator Population	66	66	63 *	70 *	62	67	67	66	60 *	72 *	56 *	71 *
	Control Crop Damage	64	62	59 *	68 *	62	64	65	62	59 *	67 *	56 *	67 *
Agree Practices Reduce Animal Welfare													
Hunting over bait	Agree/Neutral/Disagree	58 *	72 *	55 *	75 *	57 *	68 *	59 *	68 *	55 *	74 *	58 *	69 *
	I don’t know enough to respond	42 *	28 *	45 *	25 *	43 *	33 *	41 *	32 *	45 *	26 *	43 *	31 *
Captive hunt	Agree/Neutral/Disagree	63 *	74 *	59 *	78 *	61 *	71 *	60 *	73 *	58 *	78 *	60 *	73 *
	I don’t know enough to respond	37 *	26 *	41 *	22 *	39 *	29 *	40 *	27 *	43 *	22 *	40 *	27 *
Hunting in wildlife preserve	Agree/Neutral/Disagree	73	78	68 *	82 *	68 *	78 *	67 *	79 *	63 *	86 *	63 *	81 *
	I don’t know enough to respond	28	22	32 *	18 *	32 *	23 *	33 *	21 *	37 *	14 *	37 *	19 *
Trapping	Agree/Neutral/Disagree	71 *	79 *	69 *	81 *	68 *	77 *	67 *	79 *	63 *	85 *	64 *	80 *
	I don’t know enough to respond	29 *	21 *	32 *	19 *	32 *	23 *	33 *	21 *	37 *	15 *	36 *	20 *
Use of dogs	Agree/Neutral/Disagree	71 *	77 *	67 *	81 *	68 *	76 *	66 *	78 *	62 *	85 *	63 *	79 *
	I don’t know enough to respond	29 *	23 *	33 *	19 *	32 *	24 *	34 *	22 *	38 *	15 *	37 *	21 *

Note 1. In the interest of brevity, the values representing the percentage of individuals in each category who did not find the practice acceptable or who did not report the group having a high ability to ensure the welfare of hunt animals has been omitted. Because the values in each column must necessarily sum to 100, omitted variables can be calculated. For example, 88.2% of those age 45 and over reported finding hunting for food acceptable. Thus, 11.8% of respondents age 45 and over did not find hunting for food acceptable. Note 2: The numbers appear in bold when there is a statistically significant difference at the 5% level. Note 3: For the purposes of this table with respect to whether the respondent agreed that the practice reduces animal welfare, those respondents selecting very strongly agree, agree, neutral, disagree, and very strongly disagree that the surveyed practices reduce animal welfare were combined into a single category called Agree/Neutral/Disagree. Note 4: For the purposes of this table, those respondents who responded that a group had high ability or very high ability were combined into a single category and are presented in this table. The remaining categories were omitted for brevity and can be calculated according to Note 1.

**Table 4:** Presentation of statistically significant differences in hunting acceptance across familiarity with hunting (α = 0.05 denoted by “*”).

Statement	Reasons/Choice	Respondent Hunts		Respondent Knows a Hunter		Eat Game Meat Obtained through Hunting		Participate in Target Shooting		Help a Hunter Look for Wildlife Sign in Preparation for Hunting	
		No	Yes	Yes	No	Yes	No	Yes	No	Yes	No
Finds Reason to Hunt Acceptable											
Finds Reason Acceptable	Obtain Food	85 *	97 *	91 *	79 *	95 *	79 *	95 *	82 *	96 *	85 *
	Trophy Hunting	33 *	57 *	41 *	29 *	44 *	30 *	45 *	32 *	57 *	32 *
	Wildlife Population Control	70 *	84 *	78 *	60 *	85 *	60 *	83 *	65 *	88 *	68 *
	Reduce Predator Population	63 *	82 *	72 *	55 *	77 *	57 *	77 *	60 *	80 *	63 *
	Control Crop Damage	61 *	74 *	68 *	65 *	74 *	54 *	72 *	58 *	80 *	59 *
Agree Practices Reduce Animal Welfare											
Hunting over bait	Agree/Neutral/Disagree	61 *	90 *	70 *	56 *	76 *	55 *	80 *	56 *	90 *	59 *
	I don’t know enough to respond	39 *	10 *	30 *	44 *	24 *	45 *	20 *	44 *	10 *	41 *
Captive hunt	Agree/Neutral/Disagree	65 *	90 *	75 *	58 *	80 *	58 *	83 *	60 *	90 *	63 *
	I don’t know enough to respond	35 *	10 *	25 *	42 *	20 *	42 *	17 *	40 *	10 *	37 *
Hunting in wildlife preserve	Agree/Neutral/Disagree	72 *	92 *	81 *	64 *	86 *	65 *	88 *	67 *	92 *	71 *
	I don’t know enough to respond	28 *	8 *	19 *	36 *	14 *	35 *	12 *	33 *	9 *	29 *
Trapping	Agree/Neutral/Disagree	72 *	92 *	81 *	64 *	86 *	65 *	87 *	68 *	90 *	71 *
	I don’t know enough to respond	28 *	8 *	19 *	36 *	15 *	35 *	13 *	33 *	10 *	29 *
Use of dogs	Agree/Neutral/Disagree	71 *	93 *	80 *	64 *	85 *	65 *	88 *	66 *	92 *	69 *
	I don’t know enough to respond	30 *	7 *	21 *	36 *	16 *	36 *	12 *	34 *	8 *	31 *

Note 1: In the interest of brevity, the values representing the percentage of individuals in each category who did not find the practice acceptable have been omitted. Because the values in each column must necessarily sum to 100, omitted variables can be calculated. For example, 81.5% of those who do not hunt found hunting for food acceptable. Thus, 18.5% of non-pet owners did not find hunting for food acceptable. Note 2: The numbers appear in bold when there is a statistically significant difference at the 5% level. In this table, all relationships are statistically significant at the 5% level. Note 3: For the purposes of this table, those respondents selecting very strongly agree, agree, neutral, disagree, and very strongly disagree that the surveyed practices reduce animal welfare were combined into a single category called Agree/Neutral/Disagree.

**Table 5:** Logistic regression results for agreement with reasons to hunt.

Variable	Respondent Finds the Following Reason Acceptable to Hunt:			
	Food		Trophy	
	Coefficient (SE)	Marginal Effect (SE)	Coefficient (SE)	Marginal Effect (SE)
*Under 45*	−0.2827 (0.2190)	−0.0282 (0.0219)	−0.0003 (0.1560)	−0.0000 (0.0360)
*Male*	0.1431 (0.2153)	0.0141 (0.0213)	0.4011 *** (0.1520)	0.0924 (0.0348)
*College Graduate*	0.1562 (0.2392)	0.0159 (0.0251)	−0.1687 (0.1730)	−0.0393 (0.0407)
*Pet*	−0.1592 (0.2273)	−0.0155 (0.0217)	−0.3304 ** (0.1631)	−0.0770 (0.0383)
*Visited a Livestock Operation*	−0.0515 (0.2510)	−0.0051 (0.0248)	0.2952 * (0.1759)	0.0678 (0.0402)
*Visited a Fair*	0.6558 *** (0.2467)	0.0649 (0.0242)	0.0481 (0.1868)	0.0111 (0.0431)
*Respondent hunts*	10.235 ** (0.5412)	0.0886 (0.0255)	0.7746 *** (0.2226)	0.1873 (0.0550)
*Respondent doesn’t know any hunters.*	−0.6997 *** (0.2298)	−0.0752 (0.0266)	−0.3275 * (0.1744)	−0.0744 (0.0389)
*constant*	10.788 (0.3479)		−0.6079 (0.2521)	
*Log Likelihood* *Prob > Chi2* *Pseudo R2* *n*	−3000.656 0.0000 0.0665 825		−5200.335 0.0000 0.0407 825	

Note 1: For all variables the marginal effect is for discrete change of dummy variable from 0 to 1. Note 2: *p*-values: * *p* < 0.10, ** *p* < 0.05, *** *p* < 0.01.
